# Starters Experience Greater Weekly Match and Total Loads than Non-Starters in a Professional Female Soccer Team: An Exploratory Analysis Within the A-League Women’s Australian Competition

**DOI:** 10.3390/s25237290

**Published:** 2025-11-29

**Authors:** Michele Lastella, Nathan Elsworthy, Dean J. Miller, Mia Lundquist, Fabio Serpiello, Aaron T. Scanlan

**Affiliations:** 1 Appleton Institute, Central Queensland University, Adelaide, SA 5034, Australia; 2 School of Health, Medical and Applied Sciences, Central Queensland University, Rockhampton, QLD 4700, Australia; n.elsworthy@cqu.edu.au (N.E.); a.scanlan@cqu.edu.au (A.T.S.); 3 S.P.O.R.T. Research Cluster, Central Queensland University, Rockhampton, QLD 4700, Australia; 4 Adelaide United Football Club, Adelaide, SA 5007, Australia; 5 Institute for Health and Sport, Victoria University, Melbourne, VIC 3011, Australia

**Keywords:** scheduling, bench, substitute, periodization, female, football, contextual

## Abstract

Comprehensive weekly load data appears relatively absent in the literature for the professional female soccer population. This exploratory observational study quantified the weekly training, match, and total loads experienced in a professional soccer team and compared these loads according to player role. Data were collected over a full season from 22 players competing in the Australian national A-League Women’s soccer competition. Internal (session-rating of perceived exertion load [session-RPE load]) and external load (total and relative values for total and high-speed running [HSR] distance) data were acquired during on-field training sessions and matches. Players were categorized as starters (started the match) or non-starters (used as a substitute in the match) within each week. Linear mixed models and effect size analyses were used to compare weekly loads between player roles. Weekly match and total load analyses revealed higher (*p* < 0.001, *moderate*-to-*very large* effects) duration, total distance, total HSR distance, and session-RPE load in starters than non-starters. In contrast, relative total and HSR distance in matches were higher (*p* < 0.001, *moderate*-to-*large* effects) in non-starters than in starters. These data provide an initial reference for the weekly loads in this competition, highlighting disparities between player roles that should be considered when developing training and preparation plans. Given the recruitment of a single team and small sample size, future research should extend upon this study to further strengthen the evidence base in this population.

## 1. Introduction

The A-League Women’s competition is a professional soccer league for female players that was founded in 2008 (as the W-League) [1]. The competition has expanded from 8 teams in its inception to 11 teams (10 Australian teams and 1 New Zealand team) [2]. The competition attracts a rich caliber of players globally, with 19 different countries being represented among players in the 2022–2023 season [2] and several being selected for national squads for the 2023 World Cup hosted in Australia and New Zealand. Accordingly, the professionalism among A-League Women’s teams has increased substantially over the years, with many now having sport science support to generate data for more evidence-informed decisions in various areas.

Increased sport science expertise combined with advanced monitoring technologies within the A-League Women’s competition has afforded coaching staff with resources to quantify the loads of their players in training and match settings. In this regard, the physical demands imposed on players are typically measured using global positioning system (GPS) devices to represent the external load [3]. In turn, psycho-physiological measures, or the internal load, are usually measured concomitantly to identify player responses to these physical demands [4]. Accordingly, load data may support decision-making within female soccer teams for various functions like training prescription and adjustments, identifying readiness for match-play, and understanding injury risk among players [5]. Indeed, applied sport science research describing the loads placed on players is an essential initial step to understand the demands encountered relative to specific competitive contexts [5]. However, evidence concerning the typical loads experienced by players competing in the A-League Women’s competition is scarce.

To date, load-monitoring studies for the A-League Women’s competition have quantified only the external loads experienced during matches [6,7,8,9] or the internal and/or external loads encountered in small-sided games during training [6,10]. These data are typically representative of only 7–21 matches [6,7,8,9] and isolated to 3 vs. 3 to 9 vs. 9 small-sided games during training [6,10] within studies gathered during seasons from 5–15 years ago. Consequently, there is a need for comprehensive and contemporary investigation in this population. In this regard, only one study has quantified the external and internal loads experienced during individual training sessions and matches across the 2022–2023 season within this competition, highlighting sessional periodization trends within each week [11]. Consequently, limited load data have been reported for the A-League Women’s competition during training and matches across an entire season [11], with no research quantifying the accumulated weekly loads experienced. Such information is essential to comprehensively understand the current load profiles experienced in training and match settings across the A-League Women’s season—especially within weekly blocks, which is a common timeframe considered by soccer coaches and performance staff for interpreting load data and planning training microcycles [12,13,14], as well as reported in the soccer literature [15,16].

Player role (starters vs. non-starters) is an important factor to consider when quantifying load data, given systematic evidence suggests that greater match exposure in starters promotes higher loads than in non-starters among soccer players [17], potentially jeopardizing optimal training status and readiness for match-play across the entire team. However, the previous systematic review on this topic [17] highlighted only 4 of the 22 included studies examined females, with no research encompassing both external and internal load variables strictly in professional female soccer players. Comprehensive weekly load data during training sessions, matches, and in total for starters and non-starters appears absent in the literature for the professional female soccer population on the whole. Consequently, exploring this topic in players from the A-League Women’s competition seems essential and may provide useful evidence to inform decision-making among coaches and performance staff working in professional female soccer teams on pertinent aspects like training strategies and player rotation management. Therefore, the aims of this exploratory study were to (1) quantify the typical weekly training, match, and total loads experienced during the in-season in an A-League Women’s professional soccer team; and (2) compare these loads according to player role (starters vs. non-starters).

## 2. Materials and Methods

### 2.1. Participants

This exploratory research utilized a retrospective observational design whereby 22 players (age: 22.5 ± 3.7 years; height: 170 ± 7 cm; body mass: 65.3 ± 7.7 kg) from the same team competing in the A-League Women’s Australian national competition participated. Regarding player role, this sample was categorized as starters (i.e., started the match for that week) or non-starters (i.e., substitute player for that week) for each week of the in-season. To be included in the study, players had to be registered with the investigated team and participate in at least 50% of all team field-based training sessions and matches combined across the in-season. In this regard, players may have been considered as starters in some weeks and non-starters in other weeks, depending on their assigned role. In circumstances where players were unused substitutes during matches, their data were not included in the analyses for that week. Goalkeepers were excluded from analyses due to the unique demands they experience in matches compared to the remaining positions [18]. Players were monitored during all team field-based training sessions and matches across the in-season phase during the 2022–2023 season. The 20-week in-season ran from November to March, encompassing 54 field-based training sessions and 17 matches (eight at home and nine away). Overall, 149 weekly samples were collected for starters (range: 1–16) and 34 weekly samples for non-starters (range: 2–8) across players. Monitored matches took place on either a Wednesday (*n* = 1), Friday (*n* = 1), Saturday (*n* = 8), or Sunday (*n* = 7) in each week. Regarding match scheduling, no weekly matches were completed in 3 weeks, one weekly match was completed in 16 weeks, and two weekly matches were completed in 1 week across the in-season. During the two-match week, two players were removed from the analyses, as they performed different roles across these matches. This study was approved by the Central Queensland University Human Research Ethics Committee (#0000024246).

### 2.2. Procedures

The Strengthening the Reporting of Observational Studies in Epidemiology (STROBE) statement [19] was used to develop this manuscript. Players had external and internal loads monitored during each training session and match across the in-season. Training session durations were determined from the commencement of the team warm-up (given it was prescribed as part of the training stimuli) until the completion of the final drill [20]. Match durations were determined as the time each player was on the field competing between the start and end of the match, excluding any time on the bench and during the half-time break [21].

External load was measured with Catapult PlayerTek^TM^ GPS devices (PlayerTek^TM^ Pod; Catapult Sports; Melbourne, Australia; specifications: 10-hz sample rate), which were fitted to the torso of each player before each session via neoprene vests worn under training and playing attire. These devices have been used to monitor external loads in professional female soccer players [11,22], with separate field-based team sport research [23] supporting their validity (total distance vs. manual distance measurement = 349.6 ± 4.8 m for a 350-m track and 40.2 ± 2.1 m for a 40-m sprint, *p* > 0.05; peak speed vs. radar gun speed = 28.1 ± 2.1 km·h^−1^ vs. 28.9 ± 2.4 km·h^−1^, *p* > 0.05) and retest reliability (coefficient of variation = 1.1% for total distance to 11.7% for very high-speed running distance [>22 km·h^−1^]) during running and sprinting activities. It should be noted that the high-speed running threshold adopted for these previous validity data [23] supersedes the threshold we adopted tailored to the population examined (>18 km·h^−1^). Data were downloaded from each device following each training session and match for processing via accompanying software (PlayerTek^TM^ Cloud; Catapult Sports; Melbourne, Australia). Outputs for each player were trimmed with the proprietary software using manually recorded match timings of substitutions and half-time breaks, as well as visual inspection of traces [24]. External load variables taken for each session included duration (min), total distance (m), relative total distance (m·min^−1^), high-speed running (HSR) distance (distance covered at speeds > 5 m·s^−1^ or 18 km·h^−1^) [25], and relative HSR distance (m·min^−1^).

Internal load was monitored via players providing their personal session ratings of perceived exertion (RPE) with the modified Category-Ratio scale (CR-10), anchored from 0 (‘rest’) to 10 (‘maximal exertion’) to the same investigator ~30 min following each session in the absence of peers [26,27]. Session-RPE was then multiplied by session duration (min) to calculate session-RPE load in arbitrary units (AU) [26]. Data summaries were then exported as a Microsoft Excel spreadsheet (version 15, Microsoft Corp; Redmond, WA, USA) for storage and preliminary analyses. Weekly data for players undergoing adjusted training sessions due to injury or who did not complete sessions were excluded from analyses [28].

### 2.3. Statistical Analyses

All load data were imported (external load) or input manually (internal load) into Microsoft Excel and then transferred into RStudio (version 4.1.3; R Core Team). All internal and external load variables were aggregated for each player and presented across all training sessions within each week (i.e., training load), all matches within each week (i.e., match load), and all training sessions and matches combined each week (i.e., total load). All load variables were summed across relevant sessions within each week to derive weekly values for each player, with total distance and HSR distance also determined relative to the total duration in each week and expressed per minute. Load variables were determined across all players within each player role as estimated marginal means (EMM) ±95% confidence limits (CL). To examine differences in weekly training, match, and total load between player roles, separate linear mixed models were constructed for each load variable. A customized script was developed for all analyses, with models built using the *lme4* [29] package in RStudio (version 4.1.3; R Core Team). Pairwise comparisons were determined using *emmeans* [30], and model diagnostics were determined using *sjPlot* [31]. Specifically, to examine training and match loads according to player role, load type (training, match) and player role (starter or non-starter) were entered into the model (Model 1) as an interacting fixed effect (load_type * role), with player and week entered as random effects to account for the individual variability among players and match-to-match variability commonly seen within with team sport settings [32]. Various iterations of fixed and random effects were trialed and checked using the *see* [31] and *performance* [33] packages to determine the best-performing model [31]. It was determined that the full interaction model resulted in the lowest Akaike information criterion (AIC) values and was therefore selected for analyses. Histograms and Q-Q plots of the residual values were checked for each variable and were found to be normally distributed. Moreover, weekly accumulated loads were summated from training and match loads and analyzed using a separate model (Model 2), with player role entered as a fixed effect, and player and week entered as random effects. Again, this model was found to be normally distributed following the same procedure as Model 1. Statistical significance was set at an alpha level of 0.05. Hedges’ *g_av_* effects sizes (±95% CL) were also determined to quantify the magnitude of differences in variables between player roles, following recommendations by Lakens [34] using the EMM values. Effects size magnitudes were interpreted as *trivial* ≤ 0.20; *small* = 0.20–0.59; *moderate* = 0.60–1.19; *large* = 1.20–1.99; or *very large* ≥ 2.00 [35].

## 3. Results

**Figure 1:**
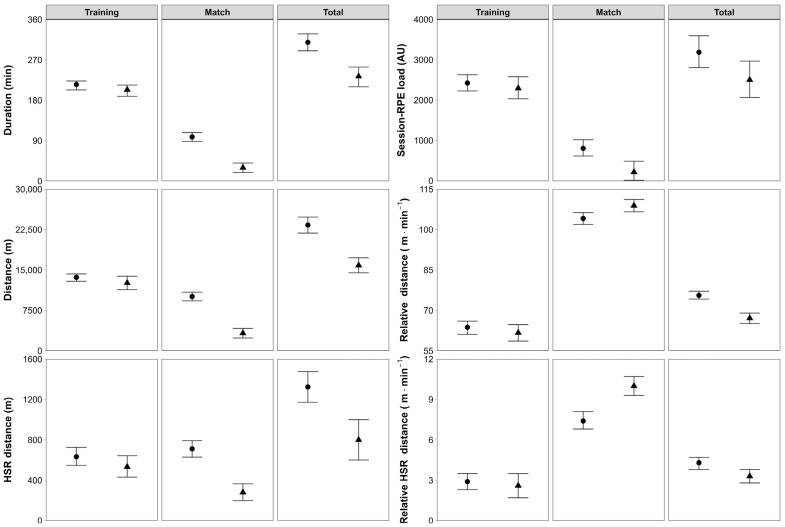
Estimated marginal means (±95% confidence limits) for weekly training, match, and total loads in A-League Women’s soccer players according to their role (circle = starters; triangle = non-starters). *Abbreviations*: RPE, rating of perceived exertion; AU, arbitrary units; HSR, high-speed running.

**Figure 2:**
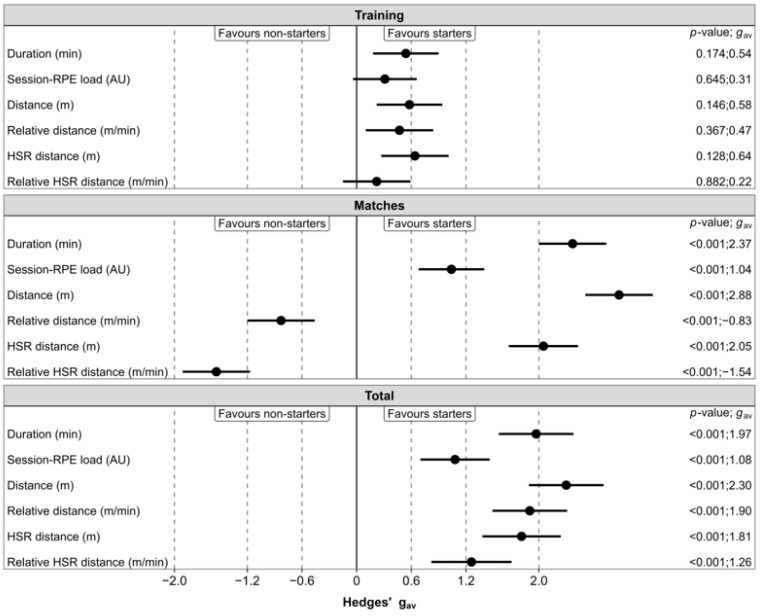
Statistical pairwise comparisons in weekly training, match, and total loads between starters and non-starters in A-League Women’s soccer players. *Note*: circles represent effect sizes, with whiskers representing accompanying 95% confidence limits; dotted lines represent cut-points for *moderate* (0.6), *large* (1.2), and *very large* (2.0) effects. *Abbreviations*: RPE, rating of perceived exertion; HSR, high-speed running.

The EMM ±95% CL for weekly load variables according to player role are presented in Figure 1, and the outcomes of the linear mixed models are presented in Table 1. Pairwise comparisons between starters and non-starters for each weekly load variable accumulated during training, matches, and in total are summarized in Figure 2.

Outcomes from Model 1 revealed that weekly internal and external loads during training were similar across player roles (*p* > 0.05, *trivial*-to-*small* effects). Outcomes from Model 1 also showed weekly match duration, total distance, and HSR distance (*very large* effects), as well as weekly match session-RPE load (*moderate* effect), were significantly (*p* < 0.001) higher in starters compared to non-starters. In contrast, weekly relative total and HSR distance during matches were significantly (*p* < 0.001, *moderate*-to-*large* effects) higher in non-starters compared to starters. Regarding weekly total loads (Model 2), all variables were significantly (*p* < 0.001) higher in starters compared to non-starters (*moderate*-to-*very large* effects).

## 4. Discussion

Our exploratory study provides the first analysis of weekly demands according to player role using a comprehensive selection of load variables in professional female soccer players, and extends upon the only study [11] reporting sessional training and match load data strictly in the A-League Women’s competition. In turn, our work provides a rudimentary initial reference point regarding weekly training, match, and total loads in the A-League Women’s competition, with some key findings regarding comparisons between player roles, including the following: (1) training load variables were comparable between roles; (2) match load variables were significantly higher in starters, except distance variables expressed relative to time, which were significantly higher in non-starters; and (3) total loads were significantly higher in starters.

Considering the magnitude of the weekly loads experienced in the A-League Women’s team we monitored, our data suggest these demands are lower or overlap with other professional female soccer players participating in European competitions. For instance, the weekly total distances across training and matches combined that we observed for starters (EMM [95% CL]: 23.3 km [21.9–24.8 km]) are considerably lower than those reported in female players (*n* = 28) competing for top-ranked teams in the Finnish national league (29.1 ± 3.3 km) [36]. However, this previous research exploring Finnish competition [36] extrapolated demands for players who were substituted out of matches (but participated for at least 60 min in matches) to represent loads across entire 90 min matches and are only indicative of an acute 3-week period at the beginning of the in-season, which may have contributed to the higher total distances compared to our data. In turn, comparable weekly total distances (21.9–24.3 km) and relative total distances (65.1–71.5 m·min^−1^) to what we observed in starters (relative total distance EMM [95% CL]: 75.6 m·min^−1^ [74.2–77.1 m·min^−1^]) were apparent across individual weeks during a 4-week monitoring period during the in-season among female soccer players (*n* = 17) competing (for at least 60 min in matches) in the Spanish Women’s First Division [37]. However, the players previously examined in this Spanish competition [37] covered less HSR distance (445–545 m) (using a lower cut-point of 16 km·h^−1^) but greater relative HSR distance (5.1–6.3 m·min^−1^) than what we observed (EMM [95% CL]: 1324 m [1172–1476 m] and 4.3 m·min^−1^ [3.9–4.8 m·min^−1^]), highlighting that nuanced variations in high-intensity running demands may be apparent between professional female soccer teams and contexts despite similar total loads.

When delving into our data more precisely according to player role, starters experienced significantly higher weekly match load volumes (total distance, HSR distance, and session-RPE load) than non-starters, which can be directly attributed to the greater weekly match exposure (i.e., duration) they received via tactical management from coaching staff. More specifically, starters exhibited 69 min (95%CL: 58–79 min) more weekly match exposure on average than non-starters, which contributed to them attaining a 588 AU (95%CL: 383–793 AU) greater session-RPE load, as well as covering 6.8 km (95%CL: 5.9–7.7 km) and 0.4 km (95% CL: 0.3–0.5 km) more total distance and HSR distance, respectively (Table 1). Considering weekly training loads were similar between player roles across variables (*p* > 0.05, *trivial*-to-*small* effects), the elevated weekly match loads directly led to significantly greater total weekly loads being experienced by starters compared to non-starters. In this regard, starters experienced a 684 AU (95% CL: 433–934 AU) higher session-RPE load and covered 7.5 km (95% CL: 6.1–8.9 km) and 0.5 km (95% CL: 0.4–0.7 km) more total distance and HSR distance, respectively, than non-starters in total on average each week (Table 1). These data suggest there was a clear imbalance in loading across player roles within the team we monitored, which was not addressed via compensatory training [38] within non-starters due to the comparable training stimuli evident across roles. Accordingly, given the effectiveness of compensatory training programs in nullifying imbalances in loading between player roles in female soccer contexts [39], the implementation of such strategies may require greater consideration in the A-League Women’s competition. Contrary to our findings on variables representing load volume, non-starters exhibited significantly higher weekly loads in variables indicative of average intensity (relative total distance and relative HSR distance) during matches. This finding seems logical and is likely attributed to the timing at which non-starters typically enter matches and the briefer match exposures they experience. In this regard, non-starters are typically substituted during the latter stages of soccer matches [40] when teams may need to strengthen their defense when leading or increase scoring opportunities when scores are level or trailing opponents, meaning they may be tactically required to consistently perform at high intensities [41]. In turn, shorter participation in matches likely imposes less accumulated physiological, psychological, and physical stress in non-starters to negate the fatigue-related declines in load outputs typically seen as match-play progresses in professional female soccer teams [42].

The distinct trends in weekly load variables we observed between player roles align with those reported in other female soccer player samples [28,43,44,45,46]. For instance, weekly session-RPE loads determined via differential scales (breath-cardiovascular, leg-musculature, and cognitive) were significantly higher during matches and in total—but not in on-field team training—across a 35-week season in professional female players (*n* = 19) competing within the Spanish Women’s First Division [28]. Likewise, when accumulated across the entire season rather than weekly, greater match and total external load volumes (total distance and HSR distance [≥15 km·h^−1^]) and internal loads (heart rate variables) were apparent in starters compared to non-starters (without any significant differences in variables during on-field team training) among collegiate, first-division (*n* = 19) [44] and third-division (*n* = 22) [43] female players. Given similar variations in external and internal load variables between player roles have also been documented for professional male soccer teams within a recent systematic review [17], our study adds to the general consensus that role-based imbalances in match and total loading accumulated across seasons may be commonplace in soccer team contexts, irrespective of player sex.

While our study provides preliminary analyses of weekly loads within the A-League Women’s competition, key limitations should be considered when interpreting our findings. Firstly, only a single team could be analyzed, which limited the sample size we were able to recruit. This limitation created an imbalanced dataset between roles, with a much smaller sample able to be gathered for non-starters, given the greater number of starters in the team. The number of weekly observations available for each role was uneven (149 for starters vs. 34 for non-starters), with several players contributing data to both roles across different weeks. This imbalance may reduce the precision of estimates for non-starters and may introduce bias in the mixed model comparisons, even though random effects were included to partially account for repeated measures. Consequently, caution is warranted when generalizing the findings to professional female players who typically occupy non-starting roles. In this way, our analyses were exploratory in nature and may not be indicative of other teams within the A-League Women’s competition, emphasizing the need for larger, multi-team studies to confirm whether our findings may be generalized across the league. Moreover, larger player samples would provide an opportunity for more nuanced analyses on this topic, such as comparisons according to playing position [47] and season phase [48], given that they impact the loads encountered by female soccer players. Secondly, contextual factors were not considered in our analyses, given that we only monitored players across a single season. Wider investigation encompassing multiple seasons may permit key contextual factors that impact the training and/or match loads experienced in soccer players, like match schedule [11], playing venue location [49,50,51,52], match outcome [49,50,53,54], and opponent strength [50,51,53]. Thirdly, our study encompassed only on-field team training sessions and matches, with off-field sessions like resistance training excluded. While this approach is commonplace when comparing loads between player roles in female soccer teams, future research should consider methods to capture wider demands, such as strain and monotony variables, and loads imposed on players across all forms of training. It is also important to acknowledge the potential measurement errors associated with GPS technology, such that the rapid accelerations, decelerations, and short-distance changes in direction typically performed in football may influence the precision of external load estimates. Finally, the understanding of, and intentions in using, collected load data among decision-makers within the team we monitored were not explored. Indeed, surveying coaches and performance staff regarding load monitoring practices within the A-League Women’s competition—and even across wider professional female soccer contexts—would provide useful insight regarding the uptake, reasoning, applications, barriers, and enablers of load monitoring in these contexts for enhanced practical impact when designing future studies on this topic [55].

## 5. Practical Applications

The need for more research in female soccer has been called for in the literature, particularly at professional levels, where the sex-based evidence gap is widest [56]. In this way, our study provides an initial reference of weekly loads within a team context for the A-League Women’s Australian soccer competition, which can be used to understand and benchmark the demands encountered. Given the increased professionalism and sport science support within teams competing in this competition, research of this nature is essential to generate data that aids evidence-informed decisions. In turn, such data may inform strategies to develop younger players across development pathways who aim to transition into the league, as well as to effectively return players from injury or extended layoffs [57].

The comparisons in load variables we conducted between player roles provide further potential to impact practice. In this regard, the significantly greater weekly load volumes during matches (~60–70% on average) led to significantly higher weekly total loads (~21–40% on average) in starters compared to non-starters, indicating a notable imbalance in the stimuli encountered across the team. This scenario may impose important ramifications whereby the lower loads experienced in non-starters may promote inferior fitness attributes in these players compared to starters across the season [58,59,60], which may reduce the level of opposition—and therefore effectiveness—that starters face during game-based drills in team training sessions. Moreover, if players who are typically non-starters undergo greater match exposure (e.g., due to injuries in starters or tactical changes in coach selection strategies and team rotations), they are likely underprepared to meet these demands acutely. Such scenarios may negatively impact team success if players possess inadequate fitness levels [58,59,60] to consistently perform at needed intensities across matches. Consequently, suitable strategies may be required to combat the imbalance in loads experienced across player roles like (1) compensatory training stimuli in non-starters via suitable drills (e.g., running-based exercises, small-sided games) [39]; (2) altered substitution strategies that reduce match exposure imbalances between player roles [61]; and (3) the introduction of a youth league or dual registration into lower competitions for increased match exposure among non-starters. Alternatively, role-specific care might be needed following matches to ensure optimal recovery in starters due to the higher loading they are faced with [17].

## 6. Conclusions

Our exploratory study adds to the scarce literature examining the loads experienced among professional female soccer players competing in the A-League Women’s competition, despite it being recognized as a prominent league on a global scale. The novel weekly load data we provide is the first of its kind for the A-League Women’s competition in the literature and therefore forms an initial dataset for reference. In turn, our analyses between player roles indicate weekly match and total load volumes were significantly higher in starters, weekly match average load intensities were significantly higher in non-starters, and weekly training loads were relatively consistent between starters and non-starters. Consequently, our findings highlight a notable imbalance in loading according to player role that could be remedied via the implementation of suitable strategies across the team.

The findings should be interpreted with caution due to the limited generalizability associated with the single-team, single-season design. In turn, this study should be viewed as exploratory and used to guide further confirmatory research. Future studies should build upon our work by incorporating data across multiple teams and seasons, allowing further integration of contextual factors into statistical models. Such broader, longitudinal approaches may enhance the understanding of load distribution and further support the development of evidence-based strategies to optimize player preparation and performance across the A-League Women’s competition.

## Figures and Tables

**Table 1:** Estimated marginal means (±95% confidence limits) for fixed effects and interactions for weekly training, match, and total load variables in A-League Women’s soccer players according to their role.

Fixed Effect	Estimate (95% Confidence Limits)					
***Model 1 (Role + Load Type)***	***Duration***	***Session-RPE load***	***Distance***	***Relative distance***	***HSR distance***	***Relative HSR distance***
Intercept	98.3 (88.4–108.2)	802 (594–1011)	10,075 (9275–10,875)	104.1 (101.9–106.3)	711 (629–792)	7.4 (6.8–8.1)
*Role*						
Starter	-	-	-	-	-	-
Non-starter	−68.5 (−79.0–−58.0)	−588 (−793–−383)	−6805 (−7696–−5914)	4.8 (2.50–7.1)	−430 (−513–−346)	2.6 (1.9–3.3)
*Load*						
Match	-	-	-	-	-	-
Training	116.9 (110.3–123.5)	1622 (1493–1751)	3552 (3015–4090)	−40.4 (−42.0–−39.1)	−78 (−126–−31)	−4.5 (−4.9–−4.1)
*Role–Load interaction*						
Non-starter × training	56.8 (41.6–71.9)	457 (160–754)	5774 (4531–7017)	−6.8 (−9.9–−3.8)	330 (220–440)	−2.9 (−3.8–−2.0)
***Model 2 (Role—Weekly total)***	***Duration***	***sRPE load***	***Distance***	***Relative distance***	***HSR distance***	***Relative HSR distance***
Intercept	309.1 (290.3–328.0)	3186 (2786–3586)	23,337 (21,851–24,823)	75.6 (74.2–77.1)	1324 (1172–1476)	4.3 (3.9–4.8)
*Role*						
Starter	-	-	-	-	-	-
Non-starter	−75.7 (−91.7–−59.6)	−684 (−934–−433)	−7473 (−8864–−6082)	−8.5 (−10.5–−6.6)	−524 (−660–−389)	−1.0 (−1.4–−0.6)

*Abbreviations*: RPE, rating of perceived exertion; HSR, high-speed running.

## Data Availability

The raw data supporting the conclusions of this article will be made available by the authors on request.

## Abbreviations

The following abbreviations are used in this manuscript:

AU	arbitrary units
CL	confidence limits
EMM	estimated marginal means
GPS	global positioning system
HSR	high-speed running
RPE	rating of perceived exertion
